# Integrating ChatGPT in Medical Education: Adapting Curricula to Cultivate Competent Physicians for the AI Era

**DOI:** 10.7759/cureus.43036

**Published:** 2023-08-06

**Authors:** Amr Jamal, Mona Solaiman, Khalid Alhasan, Mohamad-Hani Temsah, Gary Sayed

**Affiliations:** 1 Family and Community Medicine, King Saud University, Riyadh, SAU; 2 Medical Education, King Saud University, Riyadh, SAU; 3 Pediatric Nephrology, King Saud University, Riyadh, SAU; 4 Pediatric Intensive Care Unit, King Saud University, Riyadh, SAU; 5 Medical Education, California State University, Carson, USA

**Keywords:** ai & robotics in healthcare, rapid solutions, long-term solutions, artificial intelligence, curriculum adaptation, interprofessional collaboration, critical thinking, digital literacy, chatgpt, ai in medical education

## Abstract

The rapid advancements in artificial intelligence (AI) language models, particularly ChatGPT (OpenAI, San Francisco, California, United States), necessitate the adaptation of medical education curricula to cultivate competent physicians in the AI era. In this editorial, we discuss short-term solutions and long-term adaptations for integrating ChatGPT into medical education. We recommend promoting digital literacy, developing critical thinking skills, and emphasizing evidence-based relevance as quick fixes. Long-term adaptations include focusing on the human factor, interprofessional collaboration, continuous professional development, and research and evaluation. By implementing these changes, medical educators can optimize medical education for the AI era, ensuring students are well prepared for a technologically advanced future in healthcare.

## Editorial

Introduction

With the recent acceleration in the development of artificial intelligence (AI) language models like OpenAI's Chat Generative Pre-Trained Transformer (ChatGPT), drastic changes are upon us in how medical students and patients access and interact with information, as this opens new dimensions in AI integration into healthcare. Some medical experts have found ChatGPT-generated responses more valuable than Google’s search engine [1]. Although early studies have shown that ChatGPT can produce reliable information for medical students in specific fields, it is vital to confirm its standardization, reliability, and integrity [2]. This rapid development has prompted a need for medical educators to adapt their curricula and pedagogy to optimize medical students' AI competency in this evolving digital age. We suggest both short-term solutions and long-term adaptations, so medical educators can effectively navigate the AI-chatbots Large Language Model (LLM) era.

Short-term “quick fix” options

Short-term “quick fix” options are given in Table 1.

**Table 1:** Suggested short- and long-term solutions for adapting ChatGPT in medical education

Theme	Example 1	Example 2
Short-term “quick fix” solutions	Integrate digital tools into the curriculum	Teach students how to discern credible online sources
	Incorporate case-based learning	Utilize problem-solving exercises
	Focusing on evidence-based relevance	Vigilance in sorting out fake sources
Long-term longitudinal adaptations	Include empathy training in the curriculum	Teach effective communication techniques
	Organize interdisciplinary workshops	Collaborative projects with AI and data science experts
	AI-related topics in continuing education courses	Offer workshops on AI chatbot usage in healthcare
	Assess the impact of AI chatbots on students' learning	Evaluate patient outcomes related to AI-chatbot use

Promoting Digital Literacy

As a short-term solution, we recommend that medical educators ensure their students are well-versed in critical competencies of digital literacy. While digital tools are already integrated to variable extents into several medical education curricula, we encourage both faculty and students to explore novel AI technologies, like ChatGPT, cautiously, for their studies and research, ensuring they know how to critically appraise the generated information so they can discern credible sources of information from generic online platforms. While the skills needed by medical professionals for using AI in clinical settings might be similar to those required for other emerging technologies, there are crucial differences specific to transparency, health equity, and data security that set AI apart [3].

Develop Critical Thinking Skills

Educators should prioritize developing students' critical thinking skills and encourage students to question and analyze the information provided by AI chatbots. As perceptive users, students should have the capacity to recognize the limitations of LLM and verify its findings with trusted sources [2]. Incorporating case-based learning and problem-solving exercises into the medical curriculum will foster these essential skills, especially if these scenarios are empowered using AI tools [4].

Focusing on Evidence-Based Relevance

Medical students, now more than ever, need to develop competencies in recognizing what constitutes evidence-based medicine and clinical practices. The ubiquity of information generated by ChatGPT and provided by internet-based sources necessitates the development of key skills among the users. For medical students, the ability to differentiate between what constitutes valid peer-evaluated evidence as opposed to generic information made available to the public becomes ever more pressing. Moreover, in the medical field, LLMs could encounter challenges in recognizing vital information and discerning between dependable and unreliable data sources, similar to certain biases that humans might confront [5]. Medical educators must prioritize incorporating into their curriculum the significance of being vigilant about fabricated references that may be produced by LLMs [6].

Long-term “longitudinal” adaptations

Long-term “longitudinal” adaptations are given in Table 1.

Emphasize the Human Factor

While AI technologies excel at providing quick access to vast amounts of information, they traditionally cannot replace the human touch in healthcare [7]. As recent advancements have rendered LLMs more human-friendly and empathetic toward users, there is a growing need for more vigilance in their application, considering the rising potential for misinformation and its impact on users. The persuasive nature of LLMs may lead us to overlook essential human insights. A recent cross-sectional study involving 195 randomly drawn patient questions from a social media forum found that chatbot responses were preferred over physician responses. The preferred responses rated significantly higher for both quality and empathy [8]. Despite these findings, medical educators must continue to highlight human interaction and emphasize the importance of empathy, cultural competence, and effective communication in training medical students. Soft skills training should also be integrated longitudinally across the curriculum, allowing students to refine their skills throughout their education. For instance, by educating students about the limitations of chatbots in research, where LLMs might merely duplicate existing work without integrating the invaluable explorations and insights typically contributed by humans, learners can develop a deeper understanding of how to utilize these AI tools more effectively during their study years [5]. In addition, providing training to students on using LLMs as reflection tools in their critical thinking approach could be beneficial for aspiring healthcare professionals in the future [9,10].

Interprofessional Education and Collaboration

The future of healthcare is increasingly becoming integrative and collaborative between professionals, including AI experts [11]. Medical educators should create more opportunities for their students to learn from and collaborate with data scientists, computer engineers, and bioinformatics professionals. Such opportunities will not only enhance students' understanding of AI-facilitated information resources but also foster valuable teamwork skills and lead to new dimensions and transformative possibilities that may emerge from such mutually beneficial collaboration between healthcare professionals and AI experts.

Continuous Professional Development

Medical educators should promote lifelong learning and continuous professional development to stay current in the fast-paced world of AI and digital technologies. A study on creating an AI-based continuing education online training system recommended including AI-related subjects in various stages of the learning process, such as course and question bank management, learning and homework management, Q&A management, monitoring online learning, basic data management, and system management [12]. By matching the relevance of these courses, individualized learning paths can be recommended for students to optimize their continuing online training education. Integrating AI-driven approaches into professional development, continuing education, and offering workshops for medical professionals will ensure that they stay informed and adapt to the ever-evolving landscape of healthcare technology.

Research and Evaluation

Throughout the curriculum revision, medical education should continuously evaluate the effectiveness of AI-chatbot integration into the curriculum, considering the ethical implications of AI usage and prioritizing the well-being of both students and faculty. By educating themselves and their faculty on basic AI concepts and controversies, health professions education leaders can build relationships with various stakeholders such as health system informatics, clinical decision support teams, university computer science departments, and ethicists to establish a local advisory group for collaboration [13]. Researching to assess the impact of these technologies on students' learning and patient outcomes will inform future curricular developments and ensure that medical education remains responsive to the needs of the digital era.

Encourage a Holistic Approach

Emphasis on AI-based resources, like ChatGPT and other AI-Chatbots, should not be overlooked in medical education. These resources may offer various incentives for curricular enhancement, including improved personalized, self-paced, and problem-based learning experiences throughout the curriculum [14]. They can also help in improving scientific writing skills, research equity, and versatility. In healthcare research, several AI can help with the efficient and critical analysis of datasets, code generation, literature reviews, and even drug discovery and development. This allows medical students to explore such themes from the early phases of their careers. Other advantages of AI integration may consist of faculty and students’ workflow streamlining, cost savings, personalized clinical care, and enhanced health literacy, which are all integral parts of enhancing the medical education process.

Conclusion

The “ChatGPT-era” demands that medical educators reassess and adapt their teaching methodologies and curriculums while also considering the ethical implications of AI applications while maintaining a focus on the well-being of students and faculty. By implementing both short-term quick fixes and long-term adaptations, medical educators can optimize their approach to medical education, ensuring that their students thrive in a world increasingly reliant on AI technologies. Embracing the ChatGPT era is an opportunity to improve medical education and better prepare students for a technologically advanced future while engaging in national and global discussions to enhance AI training and develop AI capabilities that assist in the delivery of educational programs.
